# Comparing two large data repositories to understand the differences in demographics, health history, and behavioral attributes in populations

**DOI:** 10.3389/froh.2024.1427109

**Published:** 2024-12-04

**Authors:** Nihmath Nasiha Maliq, Toan Ong, Zachary Giano, William Rivera, Tamanna Tiwari

**Affiliations:** ^1^ School of Dental Medicine, University of Colorado, Aurora, CO, United States; ^2^ School of Medicine, University of Colorado, Aurora, CO, United States; ^3^ School of Public Health, University of Colorado, Aurora, CO, United States

**Keywords:** electronic health record, behavioral health, systemic health, health literacy, big data

## Abstract

**Introduction:**

This study conducted a comparative analysis between two large data repositories, the All of Us (AoU) medical data and BigMouth dental data repositories.

**Methods:**

The comparison analysis includes variables related to behavioral and systemic health, health literacy, and overall health status across race, ethnicity, and gender. The analytic approach used descriptive statistics, Chi-square, odds ratio, and 95% confidence intervals; significant comparisons were measured with Cohen's D effect sizes.

**Results:**

In the AoU dataset, 80.6% of Hispanic or Latino participants reported alcohol use compared to 16.8% in the BigMouth data repository. The female cohort in AoU showed 87.9% alcohol use, a contrast to BigMouth's 26.0%. Additionally, the diabetes prevalence among females was 8.8% in AoU vs. 21.6% in BigMouth. Differences in health literacy were observed, with 49.2% among Hispanic or Latino participants in AoU, in contrast to BigMouth's 3.2%. Despite this, 70.1% of Hispanic or Latino respondents in AoU reported satisfactory health status, while BigMouth indicated a much higher figure at 98.3%.

**Discussion:**

These variations highlight the importance of targeted health interventions addressing racial/ethnic and gender influences. Differences may arise from recruitment approaches, participant demographics, and healthcare access. There is a need for collaboration, standardized data collection, and inclusive recruitment to remedy these discrepancies. Further research is imperative to understand the underlying causes, facilitate interventions that address the disparities, and advocate for a more inclusive healthcare system.

## Introduction

The evolution of modern healthcare is fundamentally intertwined with data. In the digital age, the ability to gather, analyze, and interpret massive amounts of data has become pivotal for healthcare professionals and researchers (1). This data-driven approach promises to revolutionize patient care, inform public health initiatives, and optimize preventive measures (1). With this in mind, the selection and scrutiny of health datasets becomes of paramount importance (1).

The role of datasets in the healthcare paradigm is not just limited to informing decisions but is also a tool of empowerment (2). Through these datasets, a clearer picture of community health, behavior, and needs can be extrapolated. As healthcare professionals increasingly rely on electronic health record (EHR) data, the responsibility lies in ensuring its precision and reliability (3). The prominence of high-quality data stands undeniable, especially in an era where medical decisions are deeply intertwined with electronic records. With the surge in data volume, addressing the persistent shortcomings in data quality becomes indispensable, underscoring the imperative of streamlined data collection and rigorous management to fortify a proactive and informed healthcare ecosystem (2).

The present study focuses on comparing two major health datasets: All of Us (AoU) and BigMouth. Both of these datasets offer insights into diverse health parameters, encompassing behavioral health patterns, systemic health indicators, health literacy, and an overarching view of overall health status. However, as with any data source, the methodologies, sampling strategies, and intrinsic characteristics of the population under study can result in variations, nuances, and divergences in the data.

The BigMouth dataset, predominantly a dental repository, is tailored to enhance research feasibility, informatics, and quality improvements (4). Meanwhile, the AoU dataset embarks on a comprehensive mission, aiming to encompass over a million diverse U.S participants (5) that includes a wealth of data ranging from participant-provided information (PPI) from surveys to data extracted from electronic health records (EHRs), and biological samples, offering an all-encompassing view of health through its diverse data streams and sustained participant involvement (5, 6).

The rationale for comparing these two datasets is rooted in the growing recognition that oral health is closely linked to systemic health. Conditions such as type II diabetes and smoking-related illnesses have clear correlations with oral health, highlighting the need to integrate dental and medical data for a more comprehensive understanding of health outcomes. By comparing the BigMouth and AoU datasets, this study seeks to identify discrepancies in data quality, completeness, and standardization across both domains, which is essential for improving healthcare strategies and research outcomes. Integrating these perspectives can lead to more informed health strategies, enhanced public health interventions, and ultimately better overall health outcomes. In addition, evaluating these differences can enhance data integration across dental and medical repositories, fostering a more holistic approach to healthcare that bridges oral and systemic health. This comparison will shed light on potential gaps or overlaps, helping to ensure that both dental and medical health data are accurately represented in research and public health planning.

It becomes crucial, therefore, to juxtapose these datasets to discern similarities and, more importantly, differences that could potentially inform or misinform healthcare strategies. By understanding the nuances of these datasets, healthcare professionals can make informed decisions, public health officials can devise more targeted strategies, and researchers can identify gaps in current knowledge, spurring further investigation (5). Therefore, the key objectives of this study are to compare the AoU and BigMouth datasets, assess their differences in data quality and relevance, and understand how these variations may influence healthcare decisions, public health strategies, and research outcomes.

## Methods

### Data sources

Data were sourced from two repositories: BigMouth and AoU. These datasets were chosen due to their relevance and comprehensiveness in addressing the research objectives.

### AoU data

The PPI and EHR data from AoU were used. PPI is collected through surveys via the secure online AoU participant portal (6, 7). The AoU dataset is organized into three distinct tiers – Public, Registered, and Controlled tier (8). Data for this study were extracted from individual-level survey information situated within the Registered Tier.

### Data extraction

**Figure 1:**
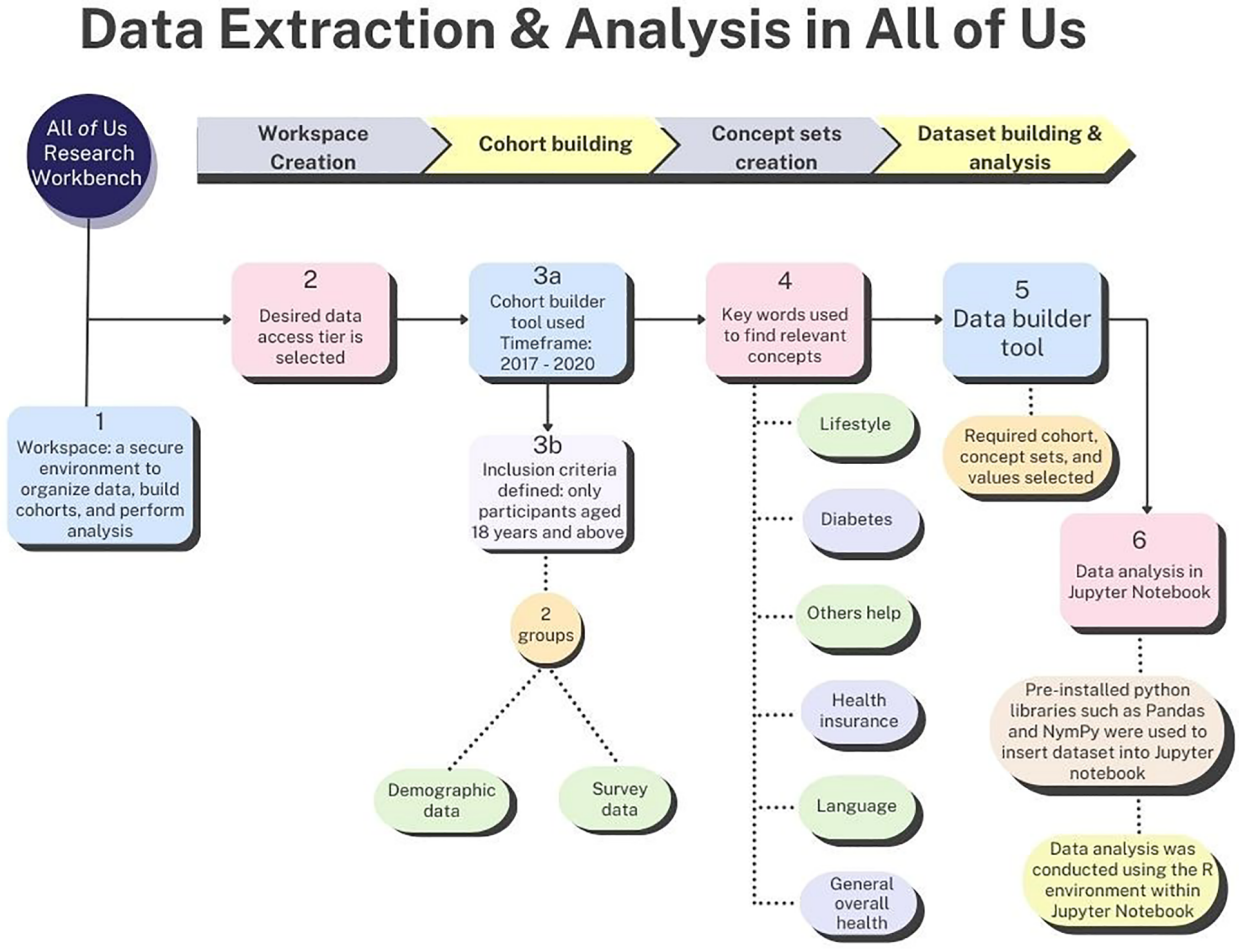
Process of data extraction and analysis in the AoU researcher workbench environment.

Figure 1 describes the data extraction process, which requires the following steps–creation of a workspace (i.e., an isolated environment in which to perform analysis on the sampled data), building a cohort, developing question concept set, dataset building, and data analysis. A workspace tailored to this study was created in the AoU research hub. Also, the desired data access tier was selected at this stage. The data utilized in this research spans the period from 2017 to 2020, offering a broad and detailed collection of surveys and EHR data for thorough analysis.

Utilizing the cohort builder tool, data were organized into a cohort comprising two categories, namely “program data,” which included demographics, surveys, and physical measurements and “domains” which included conditions, procedures, drugs, measurements, and visits (7). Only participants aged 18 years and above were included. Subsequently, a cohort consisting of two groups were constructed: one included demographic data and the other focusing on survey data. These groups were amalgamated into a single dataset, encompassing race, ethnicity, gender, tobacco usage, alcohol consumption, health insurance status, predominant household language, assistance required for reading health materials, and type 2 diabetes incidence.

Concept sets served as filters to delineate the specific rows targeted for analysis (9). Upon cohort construction, several concept sets were devised, encompassing:

• Lifestyle (tobacco participation: cigar/cigarillo, hookah, electronic nicotine, and smokeless tobacco and alcohol participation).
• Diabetes (Has a doctor or health care provider ever told you that you have? Select all that apply question with a list of endocrine conditions).
• Others help (How often do you have someone help you read health-related materials?).
• Health insurance (What kind of health insurance or health care coverage do you have?).
• Language (Do you speak a language other than English at home?).
• General overall health (In general, would you say your health is).

The AoU research platform did not permit granular selection of specific endocrine conditions (e.g., type 2 diabetes) while creating the diabetes concept set. Consequently, additional filtering of participants diagnosed with type 2 diabetes was achieved during the coding phase of the analysis.

After creating concept sets, the dataset was built using the dataset builder tool by selecting the required cohort, concept sets, and the necessary values such as diagnosis of Type 2 diabetes and responses to alcohol consumption (yes/no) (9). At the end of these processes, the dataset encompassed a total of 368,963 participants.

### Data analysis

Analysis occurred within the secure confines of the Researcher Workbench, utilizing the features of the Jupyter Notebook environment—an interactive, computational notebook used for scientific communication, data analysis, visualization, and more, depending on the code contained within the notebook. Initially, pre-installed python libraries such as Pandas and NymPy were used to insert dataset into Jupyter Notebook. This was followed by data analysis using the R environment within Jupyter Notebook. Descriptive statistical analysis was conducted. This included a univariate frequency distribution for race, gender, and ethnicity. Bivariate frequency tables were generated for race, ethnicity, and gender against several variables: tobacco and alcohol participation, type 2 diabetes presence, the requirement for others’ assistance, overall health status, type of health insurance, and use of a language other than English at home. Data analysis was performed using R version 4.2.2 software (10).

### BigMouth data

Data was also sourced from the BigMouth dental database. This database stands as one of the most comprehensive repositories of dental and systemic health information, encompassing semi-anonymized EHRs. These records detail patient demographics, medical and dental histories, treatment codes, medications, and self-disclosed health specifics from over 4.5 million individuals (11). The repository consolidates data collected from 11 dental schools (11). It should be noted that each participating dental school utilizes the Axium software for patient data entry, which is subsequently integrated into the BigMouth database. The data considered in this research extends from 2017 to 2020. The dataset included 523,857 participants.

### Data extraction

The primary dataset was procured directly from the BigMouth research management team. Subsequently, the data were categorized and systematically arranged. The dataset was subjected to coding and data cleaning and a comprehensive data dictionary was devised to bolster clarity. The analysis was conducted using the SAS statistical software suite.

### Comparison between AoU and BigMouth datasets

Among the variables of interest, pertinent questions that facilitated a comparison between the AoU and BigMouth datasets were selected. Additionally, in the BigMouth data repository, ‘tobacco participation’ encompasses participants who have engaged in the use of cigars, hookah, electronic smoking devices, smokeless tobacco, or any combination thereof. For analytical clarity, participants using any of these products were grouped together in the AoU research hub. Thus, within the “tobacco participation” column, participants were classified based on their use of any, a combination, or none of the aforementioned tobacco products.

Table 1 compares the specific questions extracted from the AoU and BigMouth datasets. This comparison provides insight into the nuances and overlaps in data collection between the two sources. The primary independent variables were the demographic factors: ethnicity (Hispanic or Latino/not Hispanic or Latino), gender (female/male), and race (White, Black, Asian, Other). The dependent variables were alcohol participation, tobacco participation, prevalence of type 2 diabetes, health literacy, and perception of overall health status.

**Table 1:** Corresponding questions under each variable from AoU and bigMouth datasets.

Variables of Interest	AoU	BigMouth
Tobacco use	Cigar smoking: cigar smoke participant	Tobacco Hx:
	Have you ever used smokeless tobacco products, even one or two times? (Smokeless products: snus pouches, skoal bandits, loose snus, moist snuff, dip, spit, and chewing tobacco)	If over 12, do you use or have you used tobacco (cigarettes, cigars, smoking, snuff, chew, bidis, electronic cigarettes, hookah)?
	Electronic smoke participant	
	Hookah smoke participant	
Alcohol use	Alcohol Participant: [In your entire life, have you had at least 1 drink of any kind of alcohol, not counting small tastes or sips? (By a “drink,” we mean a can or bottle of beer, a glass of wine or a wine cooler, a shot of liquor, or a mixed drink with liquor in it.)]	Alcohol Hx: (Do you consume alcohol?)
Overall health status	In general, would you say your health is: excellent, very good, good, fair, poor, skip.	How would you describe your overall health? Excellent, good, fair, poor.
Health literacy	How often do you have someone help you read health-related materials?	How often do you need to have someone help you when you read instructions, pamphlets, or other written material from your doctor or pharmacy?
Diabetes	Type 2 diabetes	Type 2 diabetes
Race	Race	Race
Ethnicity	Ethnicity	Ethnicity
Gender	Gender	Gender

### Data analysis

Descriptive statistical analyses were performed to determine frequency distributions for the respective variables. A Chi-Square test of independence was then employed to discern statistically significant associations between the frequencies in the datasets across varied categories. To further ascertain the relational magnitude and direction between variables in the datasets, the Odds Ratio was calculated. This metric offered insights into the probability of an event manifesting in one dataset relative to its counterpart.

The odds ratio was computed as the odds of an outcome across each demographic variable category in the AoU group divided by the odds of an outcome across each demographic variable category in the BigMouth group. For each demographic subgroup (e.g., “Hispanic or Latino”), a 2 × 2 contingency table was constructed. The rows represented the two datasets (AoU and BigMouth), and the columns represented the responses for dependent variables (e.g., Yes and No for alcohol and tobacco usage). An OR greater than 1 suggests a higher likelihood of the outcome in the AoU group compared to the BigMouth group.

Additionally, using the method specified by Chinn (12), we calculated Cohen's D to provide a measure of the effect size or the magnitude of differences between two groups. Effect sizes were interpreted based on Cohen's established benchmarks: small (d = 0.2), medium (d = 0.5), and large (d = 0.8). Larger values of Cohen's D denote greater differences between the groups.

The significance and implications of these associations were interpreted based on the OR, the 95% CI, and the calculated effect sizes.

## Results

The demographic attributes of the AoU and BigMouth datasets demonstrated significant contrasts, as outlined in Table 2. Both datasets had a higher proportion of females, with AoU at 59.4% and BigMouth at 53.4%. Hispanic/Latino individuals constituted 18.0% in AoU, significantly higher than BigMouth's 6.4%. Racial disparities were also notable, with AoU reporting 53.8% White participants compared to BigMouth's 44.3%, and 19.8% Black participants compared to 10.7% in BigMouth.

**Table 2:** Population details across AoU and BigMouth datasets.

	AoU	BigMouth
Female	59.4%	53.4%
Male	36.9%	46.4%
Other	3.8%	0.04%
Hispanic or Latino	18.0%	6.4%
Not Hispanic or Latino	77.4%	63.0%
White	53.8%	44.3%
Black	19.8%	10.7%
Asian	3.4%	6.0%
Other	3.6%	2.1%

“Other” race is another single population, more than one population, and none of these.

### Behavioral health

**Figure 2:**
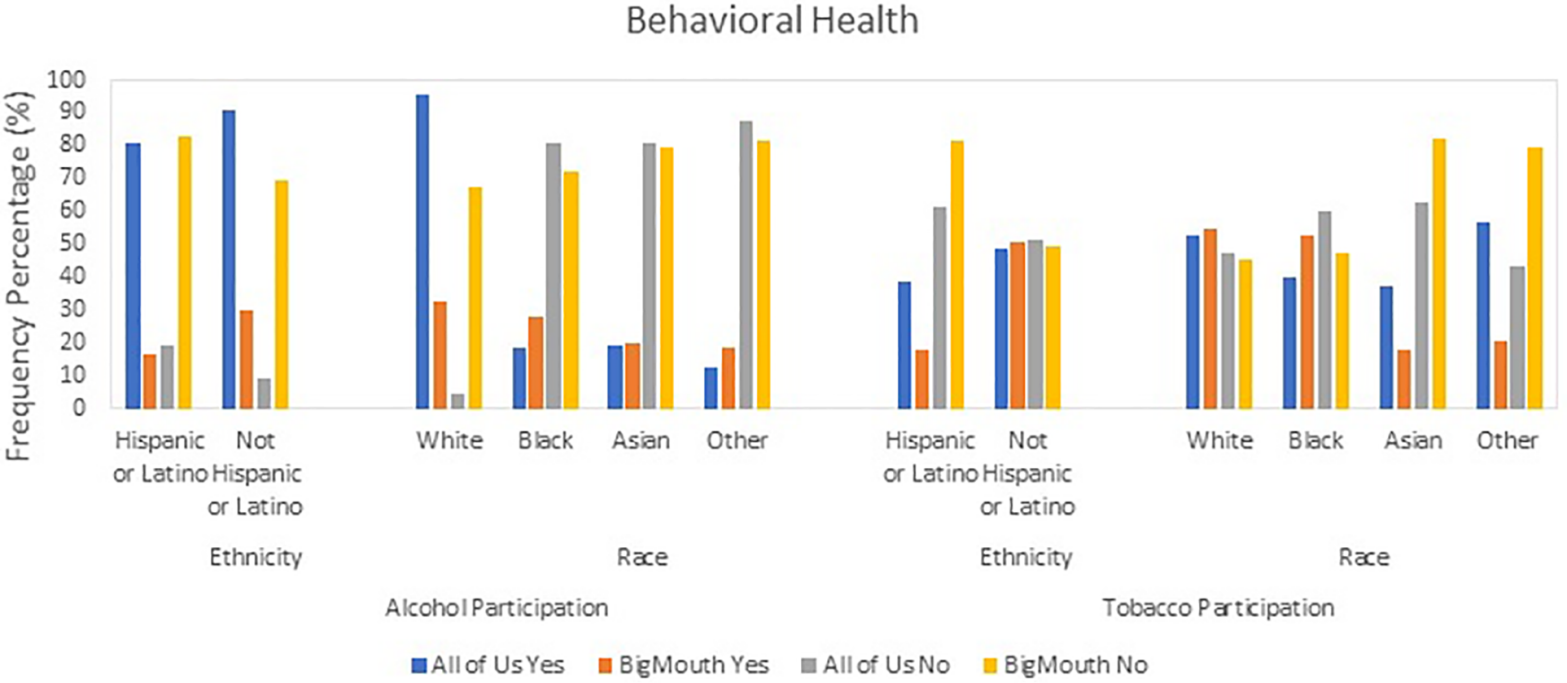
Behavioral variable health patterns: alcohol and tobacco usage in AoU vs. BigMouth.

Alcohol participation was significantly higher in the AoU dataset across all demographic groups compared to BigMouth (Figure 2). For example, 80.6% of Hispanic/Latino respondents in AoU reported alcohol use vs. 16.8% in BigMouth (OR: 20.603, Cohen's D: 1.672). Similar trends were observed in other categories, such as White respondents (95.5% vs. 32.6%, OR: 44.419, Cohen's D: 2.096) and females (87.9% vs. 26.0%, OR: 20.725, Cohen's D: 1.675) (Table 3).

**Table 3:** Comparative analysis of behavioral health, systemic health, health literacy and overall health between AoU and bigMouth datasets.

Alcohol participation			
	Odds ratio (OR)	95% CI	Effect size Cohen's D
Female	20.725	(20.36, 21.10)	1.675
Male	22.013	(21.52, 22.52)	1.708
Hispanic or Latino	20.603	(19.74, 21.51)	1.672
Not Hispanic or Latino	23.429	(23.04, 23.83)	1.743
White	44.419	(43.32, 45.55)	2.096
Black	0.605	(0.59, 0.62)	0.278
Asian	0.922	(0.87, 0.99)	0.045
Other	0.626	(0.59, 0.67)	0.259
Tobacco Participation			
Female	0.95	(0.93, 0.97)	0.028
Male	1.575	(1.55, 1.60)	0.251
Hispanic or Latino	2.854	(2.72, 3.00)	0.579
Not Hispanic or Latino	0.927	(0.91, 0.94)	0.042
White	0.924	(0.91, 0.94)	0.044
Black	0.591	(0.57, 0.61)	0.291
Asian	2.728	(2.55, 2.92)	0.554
Other	4.989	(4.73, 5.26)	0.888
Type 2 diabetes			
Female	0.352	(0.34, 0.36)	0.576
Male	0.726	(0.70, 0.75)	0.177
Hispanic or Latino	0.489	(0.46, 0.52)	0.395
Not Hispanic or Latino	0.434	(0.42, 0.44)	0.461
White	0.362	(0.35, 0.37)	0.561
Black	0.997	(0.94, 1.05)	0.001
Asian	0.431	(0.38, 0.49)	0.465
Other	0.381	(0.34, 0.42)	0.533
Health literacy			
Female	8.846	(7.04, 11.12)	1.204
Male	9.26	(7.36, 11.65)	1.23
Hispanic or Latino	29.087	(3.97, 213.31)	1.862
Not Hispanic or Latino	8.033	(6.57, 9.83	1.151
White	9.891	(7.21, 13.57)	1.266
Black	12.414	(6.74, 22.87)	1.392
Asian	5.075	(3.59, 7.59)	0.913
Other	6.913	(4.37, 10.93)	1.068
Overall health status across demographic factors			
Female	0.491	(0.47, 0.51)	0.393
Male	0.564	(0.54, 0.58)	0.317
Hispanic or Latino	0.041	(0.01, 0.17)	1.765
Not Hispanic or Latino	0.613	(0.59, 0.63)	0.271
White	0.780	(0.75, 0.81)	0.138
Black	0.441	(0.41, 0.47)	0.452
Asian	0.616	(0.55, 0.69)	0.268
Other	0.311	(0.27, 0.36)	0.645

“Other” race is another single population, more than one population, and none of these.

Tobacco participation also showed notable differences, particularly in the “Other” racial category, where 56.6% of AoU (Figure 2) respondents reported tobacco use compared to 20.7% in BigMouth (OR: 4.989, Cohen's D: 0.888) (Table 3).

### Systemic health (type 2 diabetes)

**Figure 3:**
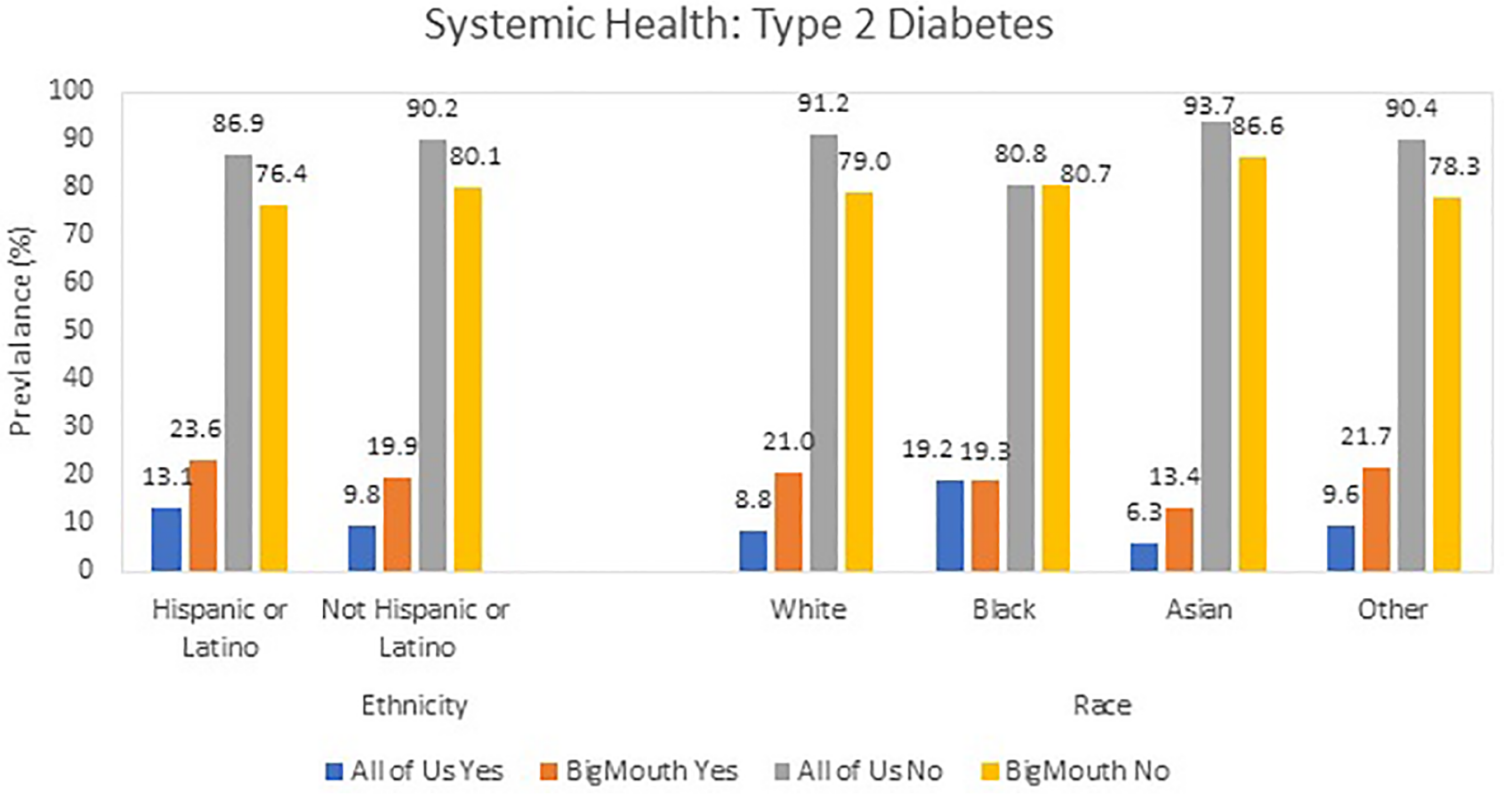
Systemic health: prevalence of type 2 diabetes prevalence in AoU vs. BigMouth.

The prevalence of type 2 diabetes differed significantly between datasets (Figure 3). In AoU, 8.8% of White participants had type 2 diabetes compared to 21.0% in BigMouth (OR: 0.362, Cohen's D: 0.56) (Table 3).

### Health literacy

AoU participants demonstrated higher rates of positive health literacy across all groups (Table 4). For example, 49.2% of Hispanic/Latino respondents reported positive health literacy in AoU vs. 3.2% in BigMouth (OR: 29.087, Cohen's D: 1.862). Similar trends were observed across racial and ethnic categories (Table 3).

**Table 4:** Health literacy metrics across demographic factors in AoU and BigMouth datasets.

	AoU					BigMouth				
	Always	Often	Sometimes	Occasionally	Never	Always	Often	Sometimes	Occasionally	Never
Hispanic or Latino	9.2%	6.7%	17.5%	15.9%	50.8%	0.0%	0.0%	0.0%	3.2%	96.8%
Not Hispanic	3.1%	3.9%	10.7%	16.5%	65.9%	0.5%	0.7%	1.1%	3.7%	93.9%
Female	3.7%	3.6%	10.6%	15.8%	66.3%	0.7%	0.6%	1.5%	2.6%	94.6%
Male	5.1%	5.7%	14.3%	17.2%	57.7%	0.4%	0.8%	1.5%	4.6%	92.7%
White	1.8%	3.1%	7.5%	17.2%	70.4%	0.1%	0.6%	0.5%	2.8%	95.9%
Black	6.8%	6.0%	19.1%	14.1%	54.1%	0.0%	0.6%	1.7%	4.1%	93.6%
Asian	2.9%	4.1%	11.7%	19.4%	61.9%	1.0%	0.3%	2.4%	6.8%	89.5%
Other	3.6%	4.7%	11.7%	17.5%	62.5%	2.0%	1.6%	1.6%	2.8%	92.0%
Overall health status measured by “Impressions of Health” perceptions across demographic factors in AoU and BigMouth datasets										
	AoU					BigMouth				
	Excellent	Very good	Good	Fair	Poor	Excellent	Good	Fair	Poor	
Hispanic or Latino	10.2%	23.2%	36.6%	23.4%	6.5%	50.9%	47.4%	0.9%	0.9%	
Not Hispanic	12.2%	32.8%	33.4%	17.5%	4.1%	12.8%	72.8%	12.8%	1.6%	
Female	11.0%	30.6%	34.6%	19.2%	4.6%	11.5%	75.2%	11.8%	1.4%	
Male	13.3%	31.7%	32.9%	17.6%	4.5%	10.4%	75.8%	12.4%	1.4%	
White	12.7%	36.3%	32.4%	14.8%	3.9%	11.5%	73.3%	13.4%	1.8%	
Black	10.5%	22.3%	36.2%	25.9%	5.1%	13.1%	70.3%	14.4%	2.2%	
Asian	15.1%	38.0%	32.9%	11.7%	2.3%	13.3%	77.6%	8.7%	0.4%	
Other	12.0%	30.6%	33.3%	18.9%	5.2%	28.7%	62.3%	7.9%	1.0%	

“Other” race is another single population, more than one population, and none of these.

### Overall health status

AoU respondents from the Hispanic/Latino group reported a satisfactory health status at 70.1% compared to 98.3% in BigMouth (OR: 0.041, Cohen's D: 1.765) (Table 3).

## Discussion

The comparison between the AoU and BigMouth datasets reveals insights into health outcomes and demographic variables. Both datasets hold intrinsic value, but highlighting their differences and commonalities is crucial for evidence-based health research and real-world applications.

In terms of behavioral health patterns, particularly alcohol and tobacco use, substantial variations were observed between the datasets. The AoU dataset showed a higher alcohol consumption rate, especially among certain demographics. This divergence could be influenced by cultural, socioeconomic, or regional factors not fully captured by the datasets. It is also possible that differences in survey methodologies contributed to these variations, as self-reported data can be subject to social desirability bias (13).

The large Cohen's D effect sizes observed (e.g., 2.096 for White respondents, and 1.672 for Hispanic/Latino respondents in alcohol participation) underscore the magnitude of the differences between the datasets and the potential for biases in data collection methodologies. These significant differences suggest that socio-cultural factors, along with the dataset methodologies, might play a role in how participants report alcohol consumption.

A notable disparity was observed in the prevalence of type 2 diabetes across the datasets. This discrepancy aligns with research by Menke et al. which delved into the varied prevalence of diabetes across racial and ethnic groups (14). While both AoU and BigMouth recorded statistically significant variations, the effect sizes were markedly different. BigMouth displayed a more pronounced effect size among Hispanic and African American populations compared to AoU, likely due to differences in sampling methodologies, population representation, or data collection strategies. For example, in the White demographic, the medium Cohen's D effect size of 0.56 highlights the extent of variation between the two datasets. These differences could result from diverse sampling methodologies, population representation, or data collection strategies, underscoring the need for further scrutiny (15).

Health literacy, a critical factor in healthcare outcomes, also presented also presented significant differences between the two datasets. Berkman et al. defined health literacy as a range of skills that enable individuals to navigate the healthcare system effectively (16). The higher rate of positive health literacy in AoU, particularly among White respondents, highlights the role of educational, socioeconomic, and healthcare access factors in shaping health outcomes. These variations highlight persistent racial disparities in healthcare, influenced by factors like healthcare access, systemic biases, and educational inequalities (17). While both datasets acknowledged the importance of health literacy in determining health outcomes, the BigMouth dataset exhibited a more pronounced association in certain groups. For example, the OR of 29.087 for Hispanic/Latino and Cohen's D of 1.862 reflect the substantial differences in health literacy across these datasets. Such differences may stem from variations in dataset composition, participant demographics, or accessibility to health resources. These findings suggest that disparities in health literacy, exacerbated by access to resources and systemic biases, can have profound impacts on health outcomes, emphasizing that health literacy is a nuanced issue that transcends mere understanding of health-related information (16).

Overall health status, as reported by respondents, differed significantly between the datasets, with AoU participants reporting a lower percentage of satisfactory health compared to BigMouth. The OR of 0.041 for the Hispanic/Latino group and the large Cohen's D value of 1.765 underline the significant discrepancies in self-reported health outcomes, which may stem from varying levels of health disparities in the two datasets. This might reflect differences in how respondents perceive their health based on their access to healthcare services or underlying health conditions (18, 19).

This study's findings highlight the potential influences of socio-demographic factors on health outcomes, an area that warrants further research. Understanding these factors is crucial for developing targeted, effective public health interventions and policies. The effect sizes and ORs for systemic health and behavioral health patterns suggest that these differences are significant enough to warrant deeper investigation into the socio-cultural and systemic factors that contribute to these health disparities. Future research should aim to understand the underlying causes of these disparities, incorporating qualitative data to capture the nuances of individual health experiences.

On a broader note, this research adds to the growing body of literature emphasizing the need for careful selection and interpretation of data sources, especially when they might be used to shape public health strategies or inform policies. Furthermore, this study boasts several strengths, notably its comparison of two extensive datasets, BigMouth and AoU, which enhances the validity and generalizability of the findings. The utilization of these datasets allows for a comprehensive analysis, given their expansive scope and diversity, encompassing varied age groups, health conditions, and socioeconomic backgrounds. Additionally, using advanced statistical methods ensures the accuracy of the results and helps account for potential confounders.

However, there are notable limitations. First, inherent biases in the datasets cannot be overlooked. As these datasets may not be entirely representative of the wider population, this could skew results or interpretations. There is also the risk of omitted variable bias, where some unobserved factors might influence the results (20). Moreover, while these datasets are vast, they are secondary in nature, which means that certain variables and nuances pertinent to the research might be absent. It is also crucial take into account the limitations faced in the categorization of racial demographics within the AoU dataset, particularly the absence of distinct categories for American Indian or Alaska Native, and Native Hawaiian or Other Pacific Islander, as outlined by the 1997 Office of Management and Budget (OMB) standards (21, 22). This limitation stems from the AoU Program's ongoing collaboration with American Indian/Alaska Native communities to ensure data integration in a responsible manner. While the Native Hawaiian or Other Pacific Islander category is present in the Controlled Tier of data (8), this study does not include that information as it relies solely on data from the Registered Tier. The key reason for this is that the Controlled Tier contains more sensitive data, such as genomic information and detailed demographic specifics, which necessitate additional approvals and heightened safeguards due to their sensitive nature (8). Given the scope of our study, which focuses on general demographic and health data, the Registered Tier data is sufficient to address our research questions without the need for the granular, sensitive information found in the Controlled Tier.

In contrast, while the BigMouth dataset initially included the American Indian or Alaska Native, and Native Hawaiian or Other Pacific Islander categories, they were merged under the “other” category for the purposes of this comparative analysis. Such consolidation should be kept in mind when interpreting findings, as it might introduce variations in the data representation. Lastly, the cross-sectional nature of the data prohibits any causal interpretations, limiting the conclusions to correlations only.

Finally, this comparison draws attention to the complexities involved in using big data for health research. The discrepancies between the AoU and BigMouth datasets emphasize the importance of considering the context in which data is collected, including participant demographics, data collection methods, and the specific objectives of each study initiative (1). Additionally, it is crucial to identify methods for secure data sharing, as open science and transparent data practices are fundamental to ensuring replicability (23). The integration of these findings with secure data practices will help ensure that dental and medical datasets are better standardized and allow for more comprehensive and actionable healthcare insights across diverse populations.

## Conclusion

The comparative analysis between the AoU and BigMouth datasets provides valuable insights into health outcomes and their demographic determinants. Notable disparities were observed in type 2 diabetes prevalence, health literacy, behavioral health patterns, and overall health status across the two datasets, emphasizing the influence of demographic factors on health outcomes. These findings highlight the importance of considering both statistical significance and effect size when interpreting health data to understand not just the probability of outcomes, but also their practical, real-world implications.

The analysis also underscores the complexities of working with large datasets in health research. The discrepancies between AoU and BigMouth demonstrate the need for careful consideration of the context, methodology, and demographic composition of each dataset to ensure accurate interpretation of findings.

## Data Availability

Publicly available datasets were analyzed in this study. This data can be found here: BigMouth Dental Data Repository (11) and All of US Data (https://allofus.nih.gov/).
